# Strategies and methods to study female-specific cardiovascular health and disease: a guide for clinical scientists

**DOI:** 10.1186/s13293-016-0073-y

**Published:** 2016-03-31

**Authors:** Pamela Ouyang, Nanette K. Wenger, Doris Taylor, Janet W. Rich-Edwards, Meir Steiner, Leslee J. Shaw, Sarah L. Berga, Virginia M. Miller, Noel Bairey Merz

**Affiliations:** Johns Hopkins University, Baltimore, MD USA; Emory University School of Medicine, Atlanta, GA USA; Texas Heart Institute, Houston, TX USA; Brigham and Women’s Hospital, Boston, MA USA; McMaster University, Hamilton, Ontario Canada; Wake Forest School of Medicine, Winston-Salem, NC USA; Mayo Clinic, Rochester, MN USA; Barbra Streisand Women’s Heart Center, Cedars-Sinai Heart Institute, Los Angeles, CA USA; Division of Cardiology, Johns Hopkins Bayview Medical Center, 301 Building, Suite 2400, 4940 Eastern Ave, Baltimore, MD 21224 USA

**Keywords:** Women, Sex-specific, Cardiovascular disease

## Abstract

**Background:**

In 2001, the Institute of Medicine’s (IOM) report, “Exploring the Biological Contributions to Human Health: Does Sex Matter?” advocated for better understanding of the differences in human diseases between the sexes, with translation of these differences into clinical practice. Sex differences are well documented in the prevalence of cardiovascular (CV) risk factors, the clinical manifestation and incidence of cardiovascular disease (CVD), and the impact of risk factors on outcomes. There are also physiologic and psychosocial factors unique to women that may affect CVD risk, such as issues related to reproduction.

**Methods:**

The Society for Women’s Health Research (SWHR) CV Network compiled an inventory of sex-specific strategies and methods for the study of women and CV health and disease across the lifespan. References for methods and strategy details are provided to gather and evaluate this information. Some items comprise robust measures; others are in development.

**Results:**

To address female-specific CV health and disease in population, physiology, and clinical trial research, data should be collected on reproductive history, psychosocial variables, and other factors that disproportionately affect CVD in women. Variables related to reproductive health include the following: age of menarche, menstrual cycle regularity, hormone levels, oral contraceptive use, pregnancy history/complications, polycystic ovary syndrome (PCOS) components, menopause age, and use and type of menopausal hormone therapy. Other factors that differentially affect women’s CV risk include diabetes mellitus, autoimmune inflammatory disease, and autonomic vasomotor control. Sex differences in aging as well as psychosocial variables such as depression and stress should also be considered. Women are frequently not included/enrolled in mixed-sex CVD studies; when they are included, information on these variables is generally not collected. These omissions limit the ability to determine the role of sex-specific contributors to CV health and disease. Lack of sex-specific knowledge contributes to the CVD health disparities that women face.

**Conclusions:**

The purpose of this review is to encourage investigators to consider ways to increase the usefulness of physiological and psychosocial data obtained from clinical populations, in an effort to improve the understanding of sex differences in clinical CVD research and health-care delivery for women and men.

## Background

Despite cardiovascular disease (CVD) being a major health-care burden and the leading cause of death in women and men in the USA, marked disparities in cardiovascular health persist between the sexes [1, 2]. Indeed, more women die annually from CVD compared with men [3]. While some studies have shown an improvement of CV outcomes in women versus men over time [3–5], others have not [6, 7]. In particular, CVD mortality is increasing in women between the ages of 35 and 54 years [8]. The reasons for this increase are controversial and reflect our limited understanding of the sex differences in physiology between women and men, which is substantially related to lack of female-specific data.

The recognition of sex differences in the pathophysiology and the expression of human disease, including CVD, led to the NIH mandate to include both women and men in clinical studies and trials and to analyze data by sex [9]. However, the number of identified variables contributing differentially to CVD outcomes in women and men is large and growing. Variables that influence CVD risk include sex chromosomes [10], hormonal status [11], disorders related to reproduction and pregnancy [12], aging, and sex and gender-specific psychological or psychosocial variables [13, 14]. Many of these variables are not considered during the design of clinical trials or longitudinal cohort studies, which reduces the ability to determine sex-specific contributors to health and disease. The lack of inclusion of sex-specific data collection also limits the ability to analyze sex differences.

The purpose of this review is to highlight variables that may contribute to our understanding of CVD in women (Table 1). We will also outline strategies and methods used in population, physiological/translational, and clinical trial research that will enable the optimization of CVD investigation and outcomes in women.

**Table 1:** Variables affecting women across their lifespan

I. Reproductive health	
Hypoestrogenemic conditions	Polycystic ovarian syndrome is associated with vascular changes [20, 229, 230]
	Stress reduces pituitary LH and FSH secretion leading to anovulation and secondary hypoestrogenemia [31, 32].
Pregnancy hypertension	A women’s recall of pregnancy hypertensive disorders is specific but sensitivity varies and the positive predictive value is low [65]. Investigators have suggested standardization of study design for research involving women with preeclampsia [231]
Maternal/fetal exposure to other pregnancy disorders	Women with histories of preeclampsia, gestational diabetes, small-for-gestational-age deliveries, or preterm deliveries (whether spontaneous or medically indicated) are at about twofold the increased risk of coronary heart disease and stroke compared with women who have had pregnancies uncomplicated by these factors [12]
Microchimerism	Fetal cells passage transplacentally into the maternal circulation during pregnancy and persist for decades (this is termed fetal microchimerism or FMC). FMC is potentially associated with detrimental effects, e.g., preeclampsia and autoimmune disease, and with beneficial effects, e.g., female longevity due to regeneration and repair due to FMC. FMC has been identified in explanted idiopathic cardiomyopathy hearts [77] and the frequency and concentration are higher in women with preeclampsia [78, 232]
Early menopause	Associated with greater coronary artery disease and stroke risk [58]
II. Sex hormones	
Endogenous sex hormones	Sex hormone levels are associated with body composition, incident diabetes, and other risk factors [233].
Hormone therapy and age of therapy	Sub-analyses from Women’s Health Initiative indicate age of hormone therapy (HT) may impact risk/benefit. The KEEPS trial showed no difference in progression of carotid intima-media thickness in women treated early post-menopause with oral or transdermal estrogen [46]
III. Psychosocial issues	
Depression	More common in women and associated with incident CVD and worse prognosis [234, 235]. The AHA has recognized depression as a risk factor for poor prognosis among patients with acute coronary syndrome [236]
Stress	Reduces pituitary LH and FSH secretion leading to anovulation and secondary hypoestrogenemia [31, 32]
Elderly age	Women are the majority of the elderly with high burden of CVD [161]
IV. Other variables	
Impact of diabetes	DM confers greater risk in women than men [171]
Non-atherosclerotic coronary disease	Vasomotor dysfunction and coronary microvascular disease are often not considered despite women having lower prevalence of obstructive CAD [34]
Inflammatory autoimmune disease	Rheumatologic disorders, particularly systemic lupus erythematosus and rheumatoid arthritis, are more prevalent in women and are associated with more prevalent CVD [176, 237, 238]

## Population research

Population research provides a unique opportunity to gather information that allows comparison of both traditional and novel CVD risk factors by sex. Previous reviews from our group and others have discussed sex and gender differences in CVD risk but have not provided guidelines for the collection of data that could expand beyond traditional risk factors [15–18]. For example, in addition to the commonly collected demographic variables, population researchers should consider including hormonal characteristics [19], sex hormonal status, pregnancy-related disorders [12], polycystic ovary syndrome (PCOS) [20], and psychosocial issues such as depression [21], abuse and domestic violence [22, 23], and post-traumatic stress disorders (PTSDs). Including this information in appropriate databases will expand the scientific understanding of the contribution of sex-related variables to sex differences in clinical CVD and increase knowledge about CVD in women and men. Data collection methods are discussed in below.

### Hormonal status

Altered menstrual patterns are a readily identifiable indicator of potential hypoestrogenism and other neurohormonal alterations. However, the causes of altered menstrual patterns are many and include both ovarian and nonovarian causes. Common ovarian causes include the following: (1) polycystic ovary syndrome, sometimes referred to as hyperandrogenic anovulation; (2) premature ovarian insufficiency which is due to reduced or accelerated loss of oocytes and is often accompanied by autoimmune disorders such as juvenile diabetes and autoimmune thyroiditis and by chromosomal abnormalities such as 45,XO or 46,XX/45,XO mosaicism or microdeletions within the long arm of the X chromosome; (3) stress-induced hypogonadism, which is accompanied by a constellation of neuroendocrine aberrations including hypercortisolism and hypothalamic hypothyroidism; (4) syndromal psychiatric syndromes such as depression, schizophrenia, and eating disorders, including anorexia nervosa and bulimia, which may alter neuroendocrine function and result in hypothalamic hypogonadism; (5) other systemic medical conditions and disorders including thyroidal conditions, hyperprolactinemia, and adrenal disorders to name but a few; (6) environmental and other unintentional exogenous hormonal exposures; (7) hormone use such as oral contraceptives; (8) drug use and abuse, including alcohol, marijuana, and opiates; (9) medications including antipsychotics and neuroleptics, selective estrogen receptor modulators (clomiphene, tamoxifen, raloxifene), and aromatase inhibitors; and (10) food-based phytoestrogens.

Women with eating disorders also often have severe nutritional compromise and significant hypercortisolemia, which may further accelerate CVD risk. There is a paucity of studies on the long-term health of women with all forms of hypothalamic hypogonadism. Greater investigative attention has been given to women with polycystic ovary syndrome due to accompanying features of metabolic syndrome

Sex differences in the molecular and physiological responses to common hormones such as cortisol and thyroid hormones likely contribute to differences in risk for stress-induced cardiovascular disease to as great an extent as hypogonadism. While stressors produce hypothalamic hypogonadism in both women and men, with attendant reductions in sex-specific hormones such as estradiol and progesterone in women and testosterone in men, the events that trigger stressful reactions differs between sexes and comparable stressors elicit different types or different magnitude responses in men and women [24]. Women may be more sensitive to certain types of psychosocial stressors than men while men appear to be more sensitive to metabolic stressors such as undernutrition than women [25–27]. During lactation, women are much less sensitive to metabolic stressors [28]. Furthermore, while stressors increase cortisol secretion in women and men, the molecular signature and other effects of cortisol upon various tissues differ by sex [29]. Cortisol antagonizes the physiological actions of estrogen through co-regulation of the same molecular pathways, suggesting that stress-induced hypogonadism cannot be counteracted by hormone replacement regimens that simply supply the missing gonadal hormones [30].

Findings from the Women’s Ischemic Syndrome Evaluation (WISE) study suggest that endogenous estrogen deficiency in young women may be a potent risk factor for atherosclerosis. While both primary ovarian insufficiency due to reduced oocyte count and/or accelerated atresia and acquired, stress-induced, ovulatory disruption result in estrogen deficiency, the latter is the most prevalent and most studied in premenopausal women. Premenopausal women with stress-induced suppression of the central drive of gonadotropic-releasing hormone (GnRH) may develop anovulation and amenorrhea or continue to have menstrual cycles but with reduced estradiol and progesterone secretion. Not only does stress have the potential to cause hypoestrogenemia, it also increases cortisol, which blocks estrogen action. Hypoestrogenemia of hypothalamic origin (defined as estradiol <184 pmol/l (50 pg/ml), FSH <10 IU/l, and luteinizing hormone <10 IU/l) was the most powerful independent predictor of angiographic coronary artery disease (CAD) such that in women with suspected ischemia, those with hypothalamic hypogonadism were at sevenfold (odds ratio = 7.4, 95 % CI of 1.7 to >30) increased risk of obstructive atherosclerotic CAD at coronary angiography [31]. Extension of this work suggests that diabetes and environmental stress may contribute to hypothalamic hypoestrogenemia with simultaneous involvement of other neuroendocrine systems [32] and inflammatory and oxidative stress mechanisms [33]. Additional studies from WISE have documented a protective association between prior oral contraceptive use during the premenopausal years and postmenopausal atherosclerosis, measured by coronary artery disease severity scores at angiography [34]. These findings combined with prior literature suggest that premenopausal estrogen deficiency due to ovarian dysfunction may be a potent risk for cardiovascular disease and may explain the more adverse prognosis experienced by premenopausal women compared to age-matched men with cardiovascular disease. However, since stress-related hypothalamic hypogonadism and other causes of hypogonadal dysfunction occur in both men and women, more research is needed to determine if there are sex differences in the frequency of hypothalamic forms of hypogonadism or if hypogonadism is more deleterious in women than in men.

Recognizing and diagnosing the cause of altered menstrual cycle function is rarely straightforward. The association between hypogonadism and cardiovascular disease may vary according to the etiology. In addition, alterations in cyclic ovarian function may affect nonreproductive tissues [35].

#### Methods for characterizing gonadal function

The first step in detecting altered ovarian function is to screen for alterations in or persistently abnormal menstrual bleeding patterns. There are many causes of abnormal uterine bleeding. An evaluation of abnormal uterine bleeding (AUB) must determine the presence or absence of endometrial and cervical polyps, fibroids, malignancy, coagulopathy, endometrial infection, adhesions, and scarring, and exogenous exposure, known and unknown, to hormones and hormonal mimetics [36]. Once the above causes have been excluded, the next step is to determine the presence or absence of ovulation and ovarian dysfunction. Ovarian patterns can be classified as fully ovulatory, anovulatory, and luteal insufficiency. There is a spectrum of ovarian function ranging from fully ovulatory to fully anovulatory. The intermediate states are the most difficult to identify, characterize, and result from a combination of wavering hypothalamic GnRH drive and varying oocyte quality. If ovarian dysfunction is identified, the cause must then be sought. Functional hypothalamic hypogonadism is likely the most common cause of AUB and amenorrhea, but it is a diagnosis of exclusion. To make matters more complex, ovarian function changes with age as the egg reserve diminishes and oocyte responses become increasingly less predictable. Classification schemes such as Stages of Reproductive Aging Workshop (STRAW) aim to partition the transition from normative ovarian function to menopause but cannot fully define an individual woman’s endogenous hormonal exposures [37]. The lack of a simple, robust methodology for classifying ovarian function and dysfunction is intrinsic to the reproductive system and likely explains, at least in part, the lack of clarity about the contribution of sex hormones to cardiovascular health.

**Fig. 1:**
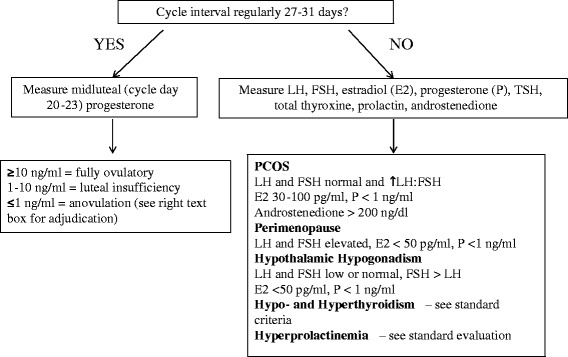
Algorithm on assessment of ovarian function

Strategies exist for characterizing alterations in gonadal function, but most of the strategies require serial blood or urine samples taken across a defined time frame often defined pragmatically as the interval between the day of first menstrual bleeding and the day of the next first menstrual bleeding. When menstrual cycles occur with predictable regular intervals, a single blood sample that is appropriately timed can allow gonadal function to be estimated [35]. For instance, in women whose menstrual cycle interval is regularly 27–31 days, the presence or absence of ovulation can be easily monitored with (1) over-the-counter urinary LH surge detector kits and (2) measuring progesterone during the midluteal phase (days 20–23 from the day of the first menses) or day +7 after LH surge. A progesterone >10 ng/ml is considered “fully ovulatory,” a level <1 ng/ml signals anovulation, and an intermediate value indicates luteal insufficiency. How often to sample blood, saliva, urine, or any other bodily tissue depends on the need to accurately define ovarian function. The more frequent the sampling, the greater the accuracy of assessment but the greater the subject burden and investigator cost. The gold standard for measurement of sex steroids such as testosterone and androstenedione in the blood or other tissue is liquid chromatography linked with mass spectroscopy. This method reduces the possibility of measuring similar steroids (greater specificity) and allows for greater sensitivity. This method may not be cost-effective or feasible in all research or clinical environments, although it is becoming more affordable and more widely available [38, 39]. Various immunoassay methodologies are commercially available [38, 40, 41]. Figure 1 provides an algorithm for assessing ovarian function.

In the systemic circulation, sex hormone-binding globulin (SHBG) binds sex steroids with greater affinity for testosterone than estrogen. This buffering mechanism keeps hormonal excursions within a given range. This mechanism does not seem to operate in the cerebrospinal fluid (CSF). Hormones, especially cortisol, in CSF are not protein bound to any significant degree, thus if hormone levels are measured in CSF, it should be recognized that these are free (bioactive) levels [42].

One potential way to mitigate the problems associated with the determination of circulating hormones is to assess such hormone concentrations in conjunction with measurement of menstrual patterns including interval between bleeding episodes and perceived flow (heavy, light, long, spotting, etc). One precautionary note is that measurement of hormones in the circulation, urine, or saliva may not be reflective of the active hormone level at the tissue or cellular level because of the local metabolism of the sex steroids. Many organs outside of the reproductive organs contain receptors for sex steroids and the exact action of sex steroids upon the various tissues of relevance are determined by tissue-specific mechanisms that may themselves differ in males and females [43]. Furthermore, interpretation of hormonal levels should be considered as the proximate integration of neuroendocrine patterns occurring at the hypothalamic level in response to central and peripheral signals. Thus, neuroendocrine function is coordinated and integrated such that there is no such condition as isolated ovarian dysfunction. Further, primary glandular diseases such as autoimmune thyroiditis alter ovarian function and compromise ovulation. The complexity is often frustrating for clinical investigators hoping for simple classification schemes.

#### Methods to identify reproductive and menopausal status

Debate continues as to whether menopausal status impacts CVD, e.g., whether observed relations are predominantly due to aging [44], to perimenopausal changes in sex hormone level status (beneficial, detrimental, or neutral) [11, 45], or to exogenous administration of sex hormones [46, 47]. Notably, sex differences in CV health and disease are present throughout the entire human lifespan not only during menarche and menopause [48]. Clearly, defining sex-specific hormonal status is crucial to the investigation into understanding CVD in women.

Menopausal status can be defined by changes in a woman’s menstrual bleeding as in the Massachusetts Women’s Health Study [49], the Study of Women’s Health across the Nation (SWAN), and the WISE study [50], among other epidemiologic studies. Following the Massachusetts Women’s Health Study [51–55], SWAN defines four menopausal status categories: *Premenopausal*, less than 3 months of amenorrhea and no increase in menstrual irregularity in the past year; *Early perimenopausal*, less than 3 months of amenorrhea with some increase in menstrual irregularity; *Late perimenopausal*, between 3 and 11 months of amenorrhea; *Postmenopausal*, 12 or more consecutive months of amenorrhea with no medical cause other than menopause. SWAN classifies women who have had a hysterectomy or a bilateral salpingo-oophorectomy (BSO) as *surgical menopausal*, a separate category from women who are naturally postmenopausal. The term “hysterectomy” needs to be carefully defined as the removal of the uterus only with preservation of the ovarian tissue as not the same as the removal of the uterus and one or two ovaries when residual ovarian function is a relevant variable. The WISE study developed an algorithm using menstrual, surgical, and reproductive history with serum hormone assays [50]. In 2011, the STRAW [37] suggested a staging system (STRAW + 10) that incorporated more recent data on hypothalamic-pituitary-ovarian function that occurs before and after the final menstrual period may be ideal [37, 50]. The type of hysterectomy (degree of ovarian preservation) or of menopause and the age of menopause have been associated with differences in risk factors and cardiovascular risk [56–58]. Therefore, these demographic data should be carefully documented in clinical trial intake information, inclusion, and exclusion criteria.

### Pregnancy-related disorders

One of the most obvious sex differences for medical research is pregnancy. While parity has been extensively studied with respect to CVD, newer research is beginning to link several pregnancy complications to increased CVD risk. While there is abundant *correlative data* of reproductive health with CVD, data are lacking regarding whether reproductive complications *cause* CVD. Even if the associations of pregnancy complications with CVD do not prove to be causal, they can serve as useful early warnings of rate of progression of subclinical to symptomatic CVD. For example, both the American Heart Association (AHA) and American College of Obstetricians and Gynecologists (ACOG) guidelines now include a history of preeclampsia as a CVD risk factor [59, 60]. The lack of comprehensive CVD and reproductive phenotyping in studies of women is a barrier to fully understanding the intimate connections between reproductive health/disorders and CVD; both fields would benefit from closer engagement and each should be considered in evaluating a woman’s CVD risk. Validity of data collected by medical records and through participant recall is discussed below.

The connections between pregnancy complications and CVD outcomes have been reviewed elsewhere [12]. In brief, women with a history of preeclampsia, gestational diabetes, small-for-gestational-age (SGA) deliveries, or preterm deliveries (whether spontaneous or medically indicated) are at about twofold the increased risk of coronary heart disease and stroke compared with women who have had pregnancies uncomplicated by these factors. Relative risks are graded by severity (earlier preterm carries higher risk than later preterm delivery) and increase with multiple complications (such as preterm preeclampsia or preterm SGA) or recurrent complications [61]. All told, between 25 and 35 % of women will experience at least one pregnancy complication during their reproductive life course [12]. Yet studies of CVD do not routinely collect detailed data on women’s reproductive histories. There are three main sources of data on pregnancy history: maternal recall, birth registry, and medical record. Each has its advantages and disadvantages, depending on the age of women studied and other contextual factors.

#### Medical record

Medical records remain the “gold standard” for characterizing a woman’s pregnancy history. Yet they have several drawbacks, including the expense of collecting and abstracting records. Primary among these is the difficulty of obtaining prenatal records for older cohorts of women, i.e., women assessed after age 60 who had pregnancies in their twenties, who are at the highest risk of CVD events over the next few years. It can be challenging to retrieve 40-year-old records, but this challenge should be reduced in the future with the use of electronic medical records.

A second difficulty is that some pregnancy complications, such as preeclampsia or the indications for an induced labor, may be recorded in part in the outpatient prenatal setting and in part in the labor and delivery records of hospitals. Finally, obstetric practice in the detection, definition, and recording of details regarding pregnancy complications has changed over time. For example, standardized testing for gestational diabetes was not routine in the USA until the 1970s, the criteria for preeclampsia and gestational diabetes have evolved over the years, and the basis of gestational dating has shifted from menstrual dating to fetal ultrasound examination. These variations in practice create challenges both for abstracting medical records for primary exposure data and for deriving a consistent definition for validation studies of birth registry data and maternal recall data. A priority goal of practical investigation is to optimize data collection of pregnancy complications relative to future CVD.

#### Birth registries

The vast national birth registries that were established in the 1960s and 1970s have been the foundation of our understanding of the links between pregnancy history and CVD. In Scandinavia, the use of a national health identification number permits the broad-scale linking of pregnancy history to CVD hospital admissions and mortality data [62]. The national registries have several advantages: population representativeness, upwards of 40 years of archived data collection, and ease of electronic data access. However, like medical records, they are subject to changes in medical definitions and practice over time. Most, if not all, studies validating birth registry data on variables such as preeclampsia, gestation length, or neonatal birth weight (used to define SGA and large for gestational age or LGA) show that most of these variables are recorded with reasonable accuracy [63, 64]. Other variables contained in birth registries and which would require clinicians or clerks to review prenatal records, such as gestational weight gain, may be less reliable. The chief drawback of relying exclusively on registry data is a usual lack of data on important covariates such as cigarette smoking during pregnancy or body mass index before pregnancy, variables which may be better collected via medical record or maternal recall.

#### Maternal recall

Given the lack of availability of medical records or the limited data fields in birth registries, investigators may wish to query mothers about their pregnancy histories. Indeed, for many pregnancy variables, maternal recall may be a truer “gold standard” than often-incomplete medical records. This may be particularly true of lifestyle factors such as smoking or drinking alcohol during pregnancy. More debatable is the ability of women to recall salient clinical details. For example, a recent review of studies of maternal recall of preeclampsia showed modest sensitivity and low predictive values of positive maternal recall compared with medical records, though specificity was high [65]. Clearly, the accuracy of maternal recall depends heavily on the degree of clinical information communicated to the mother. Currently, clinicians may share more clinical information with patients than they did 40 years ago but retention of the information if only verbal may not be accurate. The quality of maternal recall depends on exactly how women are asked about their pregnancy complication history [66, 67]. Finally, like any exposure subject to misclassification, there are methods with which to adjust exposure-disease associations for exposure misclassification, where validation studies exist to quantify the degree of misclassification [68]. There is a need for some standardization and written documentation provided to the mother regarding salient clinical features of the pregnancy that may impact her future CV risk.

Each study will have to take into account the best ways to collect data on pregnancy history, depending on the age of their cohort and the availability and quality of records and registries. The HUNT studies—a longitudinal series of clinical CVD exams in Norway—have been able to link registry data to the clinical examinations [69]. The Nurses’ Health Studies 2 has queried participants directly about pregnancy complications and is conducting medical record validation of this maternal recall [65, 70]. Other studies, such as the Framingham Heart Study [71], might combine participant recall with data from the electronic Massachusetts birth certificate. There may be pregnancy cohorts designed to study pregnancy complications, such as the US Collaborative Perinatal Project [72], that might be followed for CVD events or more recent pregnancy cohorts that can be followed for the emergence and detailed phenotyping of CVD risk variables such as vascular function. A short validated questionnaire may be a starting point to standardize these data collections [67].

One emerging risk of pregnancy relates to persistent fetal microchimerism (i.e., the persistence of fetal cells in the mother throughout life). During pregnancy, fetal and maternal cells pass across the placenta from the fetus to the mother and vice versa. These stem cells persist in the blood or tissues for decades or for the entire life of the individual [73]. Male fetal progenitor cells persist in maternal blood for as long as 27 years postpartum [74]. Microchimeric cells could have a deleterious role, inducing autoimmunity, inflammation, and neoplastic diseases. On the other hand, the stem cells could contribute to tissue repair and regeneration. Whether the sex of the fetal cells left behind in the mother plays a role in determining whether fetal microchimerism is more likely to be beneficial or harmful has not yet been clearly determined; however, it is hypothesized that HLA incompatibility may trigger a variant of chronic host versus graft disease akin to what is often seen in transplant patients [75, 76]. For example, maternal cells were found in the cardiomyocytes of male fetuses who developed neonatal heart block [77] and women who developed preeclampsia were much more likely to develop fetal microchimerism during late gestation and to have higher concentrations of fetal cells than those with uncomplicated pregnancy [78].

Ultimately, pregnancy history—including the pregnancy complications, spontaneous abortion, stillbirth, and fertility—is likely to give us new insight into sex differences in CVD. The results may prove to be clinically useful tools for illuminating new therapies and identifying the many women who might benefit from CVD preventive interventions and care tailored to their reproductive history.

### PCOS and other syndromes with altered ovarian function

Polycystic ovary syndrome (PCOS) is a common condition that affects approximately 5 % of women of reproductive age in the USA [79, 80] and is estimated to cost $4 billion annually in health-care costs [81]. Women with PCOS ovulate irregularly and can have difficulty becoming pregnant. The condition is characterized by insulin resistance and hyperandrogenemia of ovarian and possibly adrenal origin. Other organs affected by PCOS include the brain, pancreas, liver, muscle, blood vasculature, and fat [82]. In addition to reduced fertility, PCOS women have a higher incidence of diabetes, acne, obesity, hirsutism, alopecia, and polycystic ovarian morphology on ultrasound that also corresponds to higher anti-Mullerian hormone (AMH) levels and increased oocyte count. Women with PCOS are often insulin resistant and, as a consequence, may have high insulin levels, with increased risk for type 2 diabetes, high cholesterol, and high blood pressure [83]. The underlying insulin resistance is exacerbated by weight gain, obesity, and age, and women with PCOS develop metabolic syndrome earlier than ovulatory women of comparable obesity [84–87].

There remains incomplete consensus of the definition of PCOS. There are three overlapping criteria for this disorder: (1) the NIH criteria developed in 1990 at a NIH conference held to create both a working definition of the disorder and diagnostic criteria [88]; (2) the Rotterdam Criteria developed at a consensus workshop in 2003 [89]; (3) the Androgen Excess and PCOS (AE-PCOS) criteria developed in 2006 by the AE-PCOS Society [90]. All three criteria include clinical or biochemical signs of hyperandrogenism and ovarian dysfunction (chronic anovulation), while polycystic ovarian morphology is included as part of the syndrome for the Rotterdam and AE-PCOS criteria. Notably, routine medical records typically lack sufficient details to correctly identify PCOS, and self-reported PCOS was found to be inaccurate in the WISE study.

While the etiology of PCOS is multifactorial and involves excess androgen secretion by ovaries and adrenal glands (primarily androstenedione), genetic, epigenetic factors, and environmental factors, such as insulin resistance/diabetes and obesity, there is insufficient robust prospective data to clearly determine whether these abnormalities increase the incidence of cardiovascular events or other diabetic complications [91, 92]. Some studies have found evidence of greater subclinical atherosclerosis in women with PCOS compared to control cases as evidenced by increased prevalence of coronary calcium [93–95] as well as increase in carotid intima-medial thickness [96, 97]. However, these studies include relatively small numbers of women and longitudinal data are not available. Capture of data that could characterize this condition (including ovarian dysfunction and clinical or biochemical signs of hyperandrogenism, for example) in women participating in longitudinal studies designed to evaluate cardiovascular outcomes could be extremely useful and a cost-effective way to increase the scientific understanding of the chronic CVD effects of PCOS. Because there are limited data linking the general categorization of PCOS with the etiology or prevalence of clinical CVD in women, specific investigation in this area is needed.

Acquired, stress-induced, ovulatory disruption is the most prevalent cause of estrogen deficiency in premenopausal women and is discussed in detail above in “Methods for characterizing gonadal function” section. Berga et al. have showed that women with PCOS are also hypoestrogenic but not hypercortisolemic. Thus, it is important to identify the etiology of the hypoestrogenemia relative to the development of reliable and reproducible CV risk management strategies for women [98].

### Psychosocial issues

The lifetime prevalence of depressive disorders in women is approximately double that of men [99]. The incidence of depression in women is highest from puberty onward and decreases after menopause [100], with the exception of an additional spike during perimenopause [101–104]. Currently, it is not clear what causes this sex difference in mood-related disorders.

A biological susceptibility hypothesis has been suggested to account for sex differences in the prevalence of mood disorders [105, 106]. This hypothesis posits that there is a disturbance in the interaction between the hypothalamic-pituitary-gonadal (HPG) axis and other neuromodulators in women, and the neuroendocrine rhythmicity related to female reproduction is vulnerable to change and sensitive to psychosocial, environmental, and physiological factors [107, 108]. Thus, premenstrual dysphoric disorder (PMDD), postpartum depression (PPD), and mood disorders associated with perimenopause/menopause may all be related to hormone-modulated changes in neurotransmitter function [108] and should be evaluated for their relation to CVD [105, 106].

Neurotransmitter systems involved in the control of mood and behavior include the following: glutamate, gamma-aminobutyric acid (GABA), acetylcholine (ACh), serotonin (5-HT), dopamine (DA), noradrenaline (NA), and neuropeptides. Sex steroids modulate the production, uptake, and clearance of these neurotransmitters. Declining levels of estrogen in women have been associated with postpartum and post-menopausal depression, and the cyclical variations of estrogens and progesterone may be what triggers premenstrual symptoms in women [109].

Estrogen has been described as a 5-HT, NA, and ACh agonist and also modulates DA_2_ receptors [110–114]. The influence of the reproductive life cycle of women, specifically the impact of hormonal fluctuations during puberty, during the menstrual cycle, during pregnancy and postpartum, and during the perimenopause period on mood may be significant. Accordingly, age and reproductive status should be included in mood disorder clinical study and trial designs.

As mood disorders occur in both women and men, the assumption is that there must be a unified basis for their development. This common underlying cause is believed to be related to genetic predisposition. Multiple stressful life events cause biochemical changes in numerous neuroendocrine and neuroanatomical systems. As genetics determine in part how one interprets and responds to stressful life events, one may be genetically predisposed to respond in a way that leads to the development of mood disorders. The higher prevalence of mood disorders in women could be explained by an increased exposure to stressful life events, an increased vulnerability due to genetic predisposition to these events, modulation of the neuroendocrine system by fluctuating gonadal hormones, or a combination of these factors [115]. Accordingly, collection of stressful life events and reproductive/hormonal status is important for mood disorder clinical study and trial design.

Several validated and user-friendly instruments are available for the assessment and measurement of depression, anxiety, PTSD, and a history of childhood trauma. These are summarized below.

#### Depression

The Center for Epidemiologic Studies Depression Scale (CES-D) [116] is a self-report measure of depressive symptoms. The temporal stability, the specific depression severity, and factor structure of the CES-D are well established. In particular, this scale identifies negative effect, anhedonia, and somatic symptoms [117–119]. The Hamilton Rating Scale for Depression (HRSD) [120] is by far the most commonly used clinician-rated depression scale. Its reliability has been established over a period of five decades [121], and it has also been used in most studies assessing depression in cardiac patients [122, 123]. The Beck Depression Inventory (BDI) [124, 125] is a self-report assessment of depression tool. It is extremely popular amongst general practitioners and is worldwide the most used self-rating scale measuring depression [126]. Visual Analogue Scales (VAS) and their clinical applications have been established in several medical fields including depression, pain, and premenstrual mood symptoms to name a few [127–129]. As a self-rating instrument, VAS has been used to measure a variety of subjective social and behavioral phenomena; it is quick, user-friendly, literacy neutral, and in the case of depression and/or anxiety, can serve as a simple screen [130].

The two-question case-friendly instrument for depression is as good as six previously validated longer questionnaires in detecting depression in primary care [123, 131]. It asks the following:

1. “During the past month, have you often been bothered by feeling down, depressed, or hopeless?”
2. “During the past month, have you often been bothered by little interest or pleasure in doing things?”

The importance of assessing fluctuations in mood symptoms as a risk factor for CVD seems to be even more relevant in patients with bipolar disorder [132–135] as well as in dysthymia [136].

#### Anxiety

The State-Trait Anxiety Inventory (STAI) is a self-rating instrument asking about current/recent symptoms of anxiety (state) as well as how anxious the person is in general (trait) [137, 138]. It is especially helpful in treatment/follow-up studies with a focus on change over time [139, 140]. Another appropriate self-rating measure is the Generalized Anxiety Disorder (GAD) seven-item scale developed to detect GAD in primary care populations [141, 142].

#### PTSD

Several questionnaires are available for assessing PTSD. The one that includes both current and lifetime symptoms is the Clinician-Administered PTSD Scale (CAPS-1) [143]. It can be administered by both experienced clinicians and trained paraprofessionals. A more specific instrument the Harvard Trauma Questionnaire (HTQ) [144] measures torture, trauma, as well as PTSD.

#### Childhood trauma

The Childhood Trauma Questionnaire (CTQ) is the most widely used screening tool for childhood emotional, physical, and sexual abuse as well as emotional and physical neglect [145–148].

## Physiological research

There are important physiological sex differences in CVD with implications for outcomes and therapies, including coronary microvascular dysfunction, heart failure with preserved ejection fraction (HFpEF), Takotsubo cardiomyopathy, mortality due to drugs such as digoxin and QT-prolonging medications, and post-myocardial infarction (MI) depression, to name a few [15, 149]. A mounting literature documents important sex differences in pharmacology, including beta blockers, angiotensin-converting enzyme (ACE) inhibitors, and chemotherapeutic agents that have cardiovascular toxicity [150]. Accordingly, it is useful to examine the spectrum of physiological mechanisms (i.e., autonomic, metabolic, hormonal structural, and endothelial) for CVD that may differ between women and men, in order to most appropriately design sex-specific clinical studies and trials.

### Coronary microvascular dysfunction

Up to 40 % of women with signs and symptoms of myocardial ischemia presenting for invasive coronary angiography have no obstructive epicardial CAD. These women have been considered “low risk,” yet data obtained from angiographic studies demonstrate that this group includes a subgroup of patients at higher risk of adverse cardiac events [151]. Recent studies with coronary computed tomographic angiography confirm these findings in women [152, 153] and demonstrate an increased frequency of coronary vasomotor dysfunction in these women with evidence of ischemia but no epicardial coronary disease [34], consistent with coronary microvascular dysfunction. In addition, the threshold for diagnosis of impaired coronary flow reserve may differ in women and men [34, 154]. Recent studies indicate that coronary microvascular disease is also prevalent in men and impaired coronary flow reserve is a predictor of major adverse cardiac events in both men and women [155]. Ongoing investigation is addressing sex-specific mechanistic pathways for CVD in women, including traditional CVD risk factors, inflammation, and oxidative stress, leading to coronary vasomotor dysfunction and microvascular coronary dysfunction [156–160].

### Traditional CVD risk factors

A large body of evidence supports the role of traditional CVD risk factors with endothelial-dependent dysfunction and an associated adverse prognosis in women [34, 161]. However, non-endothelium-dependent microvascular dilation appears to be involved in functional and structural alterations that lead to impaired coronary flow reserve with aging [162], hypertension [163, 164], diabetes [165, 166], dyslipidemia [167, 168], and insulin resistance [169]. Adenosine increases coronary flow predominantly by non-endothelium-dependent mechanisms via receptors on microvascular smooth muscle cells to modulate intracellular Ca^2+^ [170], while flow or acetylcholine results in vasodilation by endothelium-dependent mechanisms [34]. Because coronary microvascular dysfunction appears to be more prevalent in women, these findings support that clinical study and trials should incorporate sex-specific measures and thresholds in clinical study and trials.

#### Diabetes mellitus

Diabetes mellitus is a relatively greater risk factor for CVD in women compared to men [161, 171]. Diabetes is associated with microvascular dysfunction in noncardiac organs such as the eye, kidney, and brain. Chronic hyperglycemia is associated with significantly reduced endothelium-dependent and endothelium-independent coronary vasodilator function [172]. There is evidence implicating similar adverse effects from insulin resistance or hyperinsulinemia [173] while interventions aiming at improving insulin sensitivity improve endothelial function and decrease myocardial ischemia in patients with no obstructive CAD [173]. However, other studies demonstrate that hyperinsulinemia or insulin resistance is not associated when other confounding factors are excluded [174]. Because many of these studies have not been conducted specifically in women, further investigation exploring the mechanisms underlying potential sex differences in metabolism, diabetes, and CVD is needed including studies of the sex-specific efficacy of pharmaceuticals to manage insulin resistance and their potential to interact with estrogen responsiveness to modulate CV risk.

#### Chronic inflammation

Women suffer disproportionately from autoimmune disorders compared to men. A rise in C-reactive protein (CRP) is noted in girls but not in boys at puberty [34]. High levels of high sensitivity CRP (hsCRP), a marker of low-grade chronic inflammation, were associated with increased frequency of ischemic episodes, detected by ambulatory ECG, in adults [175]. Inflammatory processes are associated with increases in oxidative stress. In patients with systemic lupus erythematosus (SLE), who are predominantly female [176], there is a 44 % prevalence of coronary microvascular dysfunction. These observations suggest that chronic inflammation related to selected endothelial cell activation may contribute to the microvascular abnormalities in SLE [177]. Investigation is needed to further explore sex-specific immune response, for example, as measured by inflammatory cytokines, and whether this response is affected by hormonal status or other sex-specific variables.

#### Novel cardiovascular risk factors

Overall, traditional CVD risk factors associated with coronary microvascular dysfunction account for <20 % of the observed variability in WISE women [178]. Therefore, other, as yet unidentified, factors must primarily account for non-endothelium-dependent coronary microvascular reactivity. Promising candidates include, but are not limited to, sex differences in the cardiac autonomic nervous system [179], regulation of nitric oxide metabolism and inflammatory cytokines, polymorphisms in estrogen or androgen receptors, and alterations in the expression or production of local vasoactive substances and their regulators such as angiotensin II, endothelin, vasopressin and their metabolites, regulators of the cyclooxygenase pathway, the reactive intermediates of that pathway, and associated intracellular signaling pathways, as well as steroid hormones as discussed above [180]. Additional investigations regarding physiological sex differences in the gut microbiome, infections, and chronic inflammatory disease, such as rheumatoid arthritis, which preferentially impact women, and sex-specific influence of periodontal disease, are needed.

### Biomarkers

Clinically, cardiovascular biomarkers are substances that are measured most typically in the bloodstream when CVD is present and help diagnose, risk stratify, monitor, and assist in the management of people with suspected acute coronary syndrome (ACS), acute MI [181], and heart failure.

In 2007, the National Academy of Clinical Biochemistry (NACB) and International Federation of Clinical Chemistry (IFCC) committee recommended that sex-specific reference limits should be used in clinical practice for some cardiac biomarkers [182], yet to our knowledge, most clinically used assays and laboratories fail to do this. The failure to establish sex-specific, clinically applicable cardiac biomarker thresholds in men and women may contribute to the under-diagnosis of CVD in women, which in turn likely contributes to lower levels of treatment and resultant elevated death rates.

The most commonly used biomarkers in CVD stratification, cardiac troponin I (cTnI), and cardiac troponin T (cTnT) have been shown to have significantly different mean concentrations between men and women, suggesting the need for establishment of sex-specific ranges [183]. In a 2015 study, Shah et al. [184] reported that when the current practice threshold for cTnI of 50 ng/L was utilized, men were twice as likely as women to be given a diagnosis of myocardial infarction (MI) despite a similar proportion of men and women reporting chest pain and demonstrating electrocardiographic changes. Using a high sensitivity assay with sex-specific diagnostic thresholds (34 ng/L for men, 16 ng/L for women) doubled the diagnosis of MI in women, such that the diagnosis proportion was now similar to men. Subsequently, they found that the women identified using high sensitivity cTnI sex-specific thresholds had the highest risk of mortality or recurrent MI, indicating that these women can potentially greatly benefit from reclassification and treatment [184]. These data combined with the many other CVD biomarkers used clinically as well as for research purposes [185] strongly supports the use of sex-specific biomarker thresholds in guidelines, practice, and research.

### Heart failure with preserved ejection fraction

Heart failure has emerged as a new CVD epidemic related in part to effective therapies for acute coronary syndromes and myocardial infarction that have reduced ischemic heart disease mortality but have increased the number of patients living with ischemic cardiomyopathy. In addition, heart failure is prevalent in older individuals, a growing segment of the population. However, half of patients hospitalized for heart failure have normal left ventricular ejection fraction [186–188]. Heart failure with preserved ejection fraction (HFpEF) has emerged as the dominant form of HF in aging populations [188, 189]. In a US Medicare population, women have a 71 % higher prevalence of HFpEF than men independent of clinical conditions that could result in pressure overload, such as hypertension or renal insufficiency [190]. HFpEF (defined as left ventricular end-diastolic pressure (LVEDP) >16 mmHg with normal ejection fraction [191]) was identified in 67 % of clinically stable outpatients with unexplained dyspnea, of whom 75 % were females [192]. Echocardiographic and brain natriuretic peptide (BNP) are used to establish a diagnosis of HFpEF. However, up to 30 % of patients who meet all other criteria for HFpEF may have normal BNP levels. The variables predictive of normal BNP in setting of HFpEF include being female, obese, and coronary artery disease [193]. LVEDP is typically measured in resting and overnight-fasting conditions and may underestimate the true prevalence of LV diastolic dysfunction as studies have demonstrated that assessment during stress increases detection of HFpEF [194].

There is currently no effective therapy that has been shown to reduce mortality in patients with HFpEF. This may be due in part to under-representation of women in heart failure clinical trials and to a lack of understanding of sex-specific heart failure pathophysiology. Cardiac remodeling differs between men and women [195, 196], and there appear to be differences in the signaling pathways regulating myocardial hypertrophy [197–199]. Clinical and basic science studies and trials for HFpEF must be designed with these issues in mind to include women and the elderly, as well as to study sex-specific mechanistic pathways.

### Takotsubo cardiomyopathy (stress cardiomyopathy)

Transient systolic and diastolic dysfunction occurring in the absence of coronary atherosclerotic disease was first described in 1990 in Japan. The disorder has a significant female preponderance. The International Takotsubo Registry published data from 25 cardiovascular centers in nine countries, including Europe and USA, showing that 89.8 % of patients were women with a mean age of 66.8 years [200]. Patients frequently present with chest pain, dyspnea, and less commonly, syncope. Physical triggers (including acute respiratory failure, post-surgical/fracture, central nervous system condition, and infections) were present in 36 % of patients, emotional triggers (including grief/loss, panic/fear/anxiety, interpersonal conflict, and anger/frustration) were present in 27.7 % patients. No evident triggers were present in 28.5 %. Neurologic or psychiatric disorders were present in 57.8 % of patients with Takotsubo cardiomyopathy as compared to 27.5 % of patients with acute myocardial infarction. It is not understood why there is such a sex difference in this disorder. It has been proposed that this may be due to sex differences in interactions between vagal-sympathetic activity and sex hormones [201].

## Clinical trials

Death due to CVD in females emerged rapidly as an epidemic in 1984 and persists today [202, 203] despite an overall decline in CVD death rates starting in the 2000s. Both an excess of death due to CVD, and an absence of understanding of the root causes of CVD in women, remains. Recommendations to improve the evidence base for women are listed in Table 2 and discussed further below.

**Table 2:** Recommendations to improve the evidence base for women with CVD [16]

**1) Improve trial design**	
a) Power trials to test heterogeneity in outcomes by sex	
b) Explore further such heterogeneity when identified	
c) Form a statistics working group to develop alternative statistical methods	
**2) Better enrollment in trials**	
a) Increase use of proven recruitment and retention strategies	
b) Research to understand sex-related differences in recruitment and retention of subjects and how to overcome them	
c) Employ regulatory and reimbursement strategies	
d) Better Centers for Medicare and Medicaid Services coverage of trial expenses	
**3) Mandate reporting of primary and secondary results in clinical trials by sex**	
a) Journal editors require sex-specific reporting in all primary manuscripts	
b) Publish or web post brief secondary presentations and/or papers on results in women	
c) Explore alternative ways to enhance accessibility of new and existing data for review and for incorporation into meta-analyses	
**4) Create incentives to enhance the performance of research in women**	
a) Alter pre-market investigational paradigm regarding sex-based data	
b) Identify business incentives	
c) Implement new FDA policies requiring discussion of the impact of sex before devices or drugs receive approval	
d) Consider extensions in patent duration for enhanced pre-clinical testing of drugs and devices in women	
e) Increase awareness of the problem among investigators, industry, and regulators	

### Recruitment, enrollment, and retention of women

**Fig. 2:**
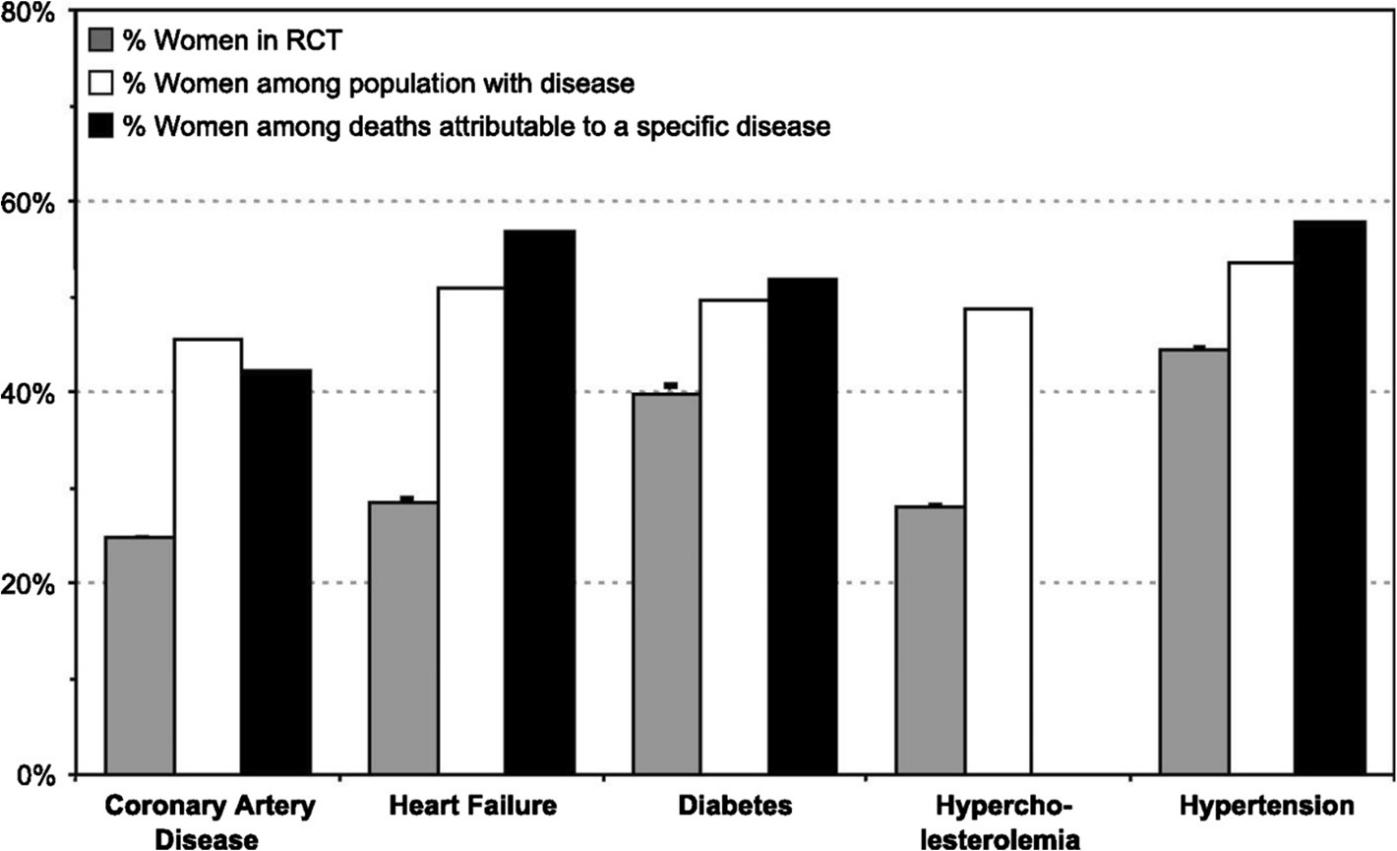
Women are underrepresented in cardiovascular clinical trials [204]

Women (minority women in particular) are underrepresented in cardiovascular clinical trials [204] (Fig. 2). Basic and clinical research often ignores the possibility of sex-related differences when designing and interpreting studies. Inclusion of women and sex-specific reporting of outcomes in clinical research remains a significant concern. An initial review of the newly mandated Federal Drug Administration (FDA) snapshots demonstrates that newly approved drugs include approximately only one-third women subjects [205]. Of great concern is that a majority of medications withdrawn following FDA approval are due to unanticipated adverse effects in women [206].

In 1986, the National Institutes of Health (NIH) established a policy for inclusion of women in clinical research, and the 1987 NIH guide encouraged the inclusion of minorities in clinical research studies. Congress transformed what previously had been policy into Public Law in the NIH Revitalization Act of 1993 (PL 103-43), which reinforced existing NIH policies but with four major differences, including (1) inclusion of women and ethnic minorities, (2) valid representation of same, (3) precludes cost exclusion, and (4) initiates recruitment and retention activities (Table 3). Subsequent activity included the establishment of a NIH tracking and inclusion committee as a monitoring group because the 1990 General Accounting Office (GAO) found that NIH policy on inclusion was inconsistently applied and had not been well communicated or understood within NIH or the external research community. Additional actions were implemented in 2001 and included (1) conversion of guidelines regarding inclusion of women and minorities into policy; (2) new terms and conditions for phase III clinical trials; (3) incorporation of these into NIH solicitations, applications, and contracts; and (4) incorporation into application reviews. As part of the continuing implementation and monitoring activities, a new enrollment/recruitment form that documented subjects’ gender became mandatory in May 2005 and required the revised categories to be used when reporting racial and ethnic data. A 2011 monitoring compliance report details the population analysis data required for both extramural and intramural research [207]. Thus, although the NIH Guidelines are not new and continued monitoring of adherence to the NIH policy is underway, the numbers of women in CVD clinical trials alongside men remains low. In October 2015, the General Accounting Office reported its findings on women’s participation in NIH-funded clinical trials as a result of a performance audit from September 2014 to October 2015 [208]. The GAO reported improvement in inclusion of women over the past two decades. However, it was recommended that the NIH provide more detailed institute and center level enrollment data and examine approaches for aggregating more detailed enrollment data at disease and condition level and to collect data on monitoring and reporting plans for sex-difference analyses.

**Table 3:** NIH Revitalization Act of 1993 (PL 103-43)

The act reinforced existing NIH policies but with four major differences:	
1. That NIH ensure that women and minorities be included in all clinical research	
2. That women and minorities be included in phase III clinical trials in numbers adequate to allow for valid analyses of differences in the intervention	
3. That cost is not allowed as an acceptable reason for excluding these groups	
4. That NIH initiates programs and support for outreach efforts to recruit and retain women and minorities as participants in clinical studies	

**Fig. 3:**
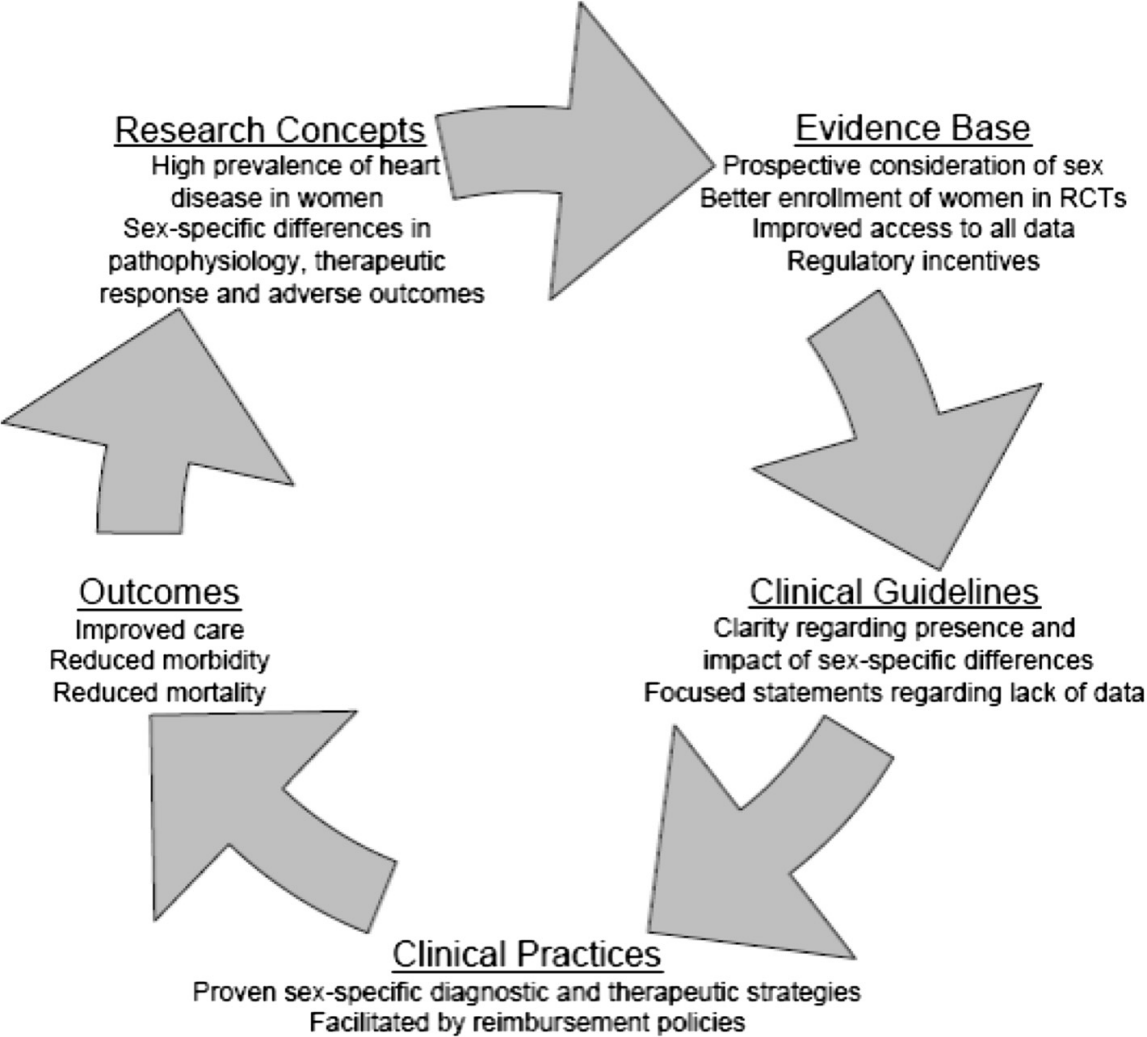
Cycle of quality to improve the care of women with CVD

Development of strategies to improve the quality of health care for women with CVD has been identified and recommendations are made [16]. Key components to improve quality care are depicted in Fig. 3 and include (1) enhance the quantity and quality of evidence-based medicine to guide care in women through improvements in trial design, enrollment and retention of women subjects, results analysis and reporting, and better incentives to perform research in women; (2) provide incentives to develop better data in women through mandating changes in the drug and device development and approval processes; (3) incorporate specific recommendations for women into guidelines when data are sufficient; and (4) apply proven sex-based differences in risk stratification, diagnostic testing, and drug usage and dosing in clinical care [16].

### The elderly (dominantly women)

Women constitute the majority of the Medicare population and an even greater percentage of very elderly individuals. Incidence and prevalence of CVD increases with aging but particularly for women who develop clinical manifestations of CVD at later age than their male counterparts (mean age for presenting with ACS for men 69 years, women 72 years) [202]. Adults age ≥75 comprise fewer than 10 % of the US population yet constitute almost half of all patients with acute coronary syndromes and over two-thirds of all acute deaths from coronary disease. Exclusion or underrepresentation of elderly individuals in clinical trials doubly disadvantages elderly women, who have the highest incidence, prevalence, and adverse outcomes of acute coronary events [200–211]. Yet, in several large registries and a clinical trial [212], older patients treated with revascularization procedures versus medical therapy had a greater risk reduction in mortality than those younger than age 65, despite their multiple comorbidities [213, 214]. Of importance, age-related pharmacokinetic and pharmacodynamic changes can alter drug dosing, efficacy, and safety and this is often compounded by polypharmacy [215, 216] further disadvantaging women. Elderly women are the group least likely to be referred to cardiac rehabilitation, where exercise can improve their functional status and secondary prevention modalities can be reinforced [217, 218]. Elderly women have the most adverse outcomes from atrial fibrillation, particularly stroke, yet anticoagulant therapy is paradoxically underutilized. In the CHA_2_DS_2_-VASc score for risk stratification, age >75, age 65–74, and female sex all define increased risk [219].

Outcomes of cardiovascular clinical trials emphasize mortality benefit, with inattention to outcomes valued by many elderly patients—independence, decreased symptoms, improved functional capacity, and decreased hospitalizations. Clinical trials in elderly patients must incorporate indices of physical and cognitive function, consider comorbidities and polypharmacy, and address outcomes desired and valued by elderly patients [220].

### Data maintenance and reporting by sex

**Fig. 4:**
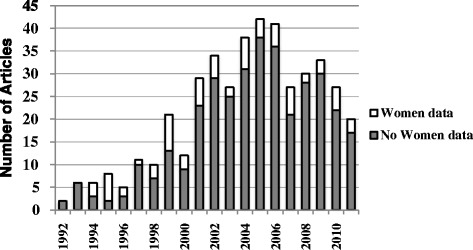
Number of articles reporting data on women by year [221]

While women have been, and remain under-represented in clinical trials [204], a great deal of data exist, but are not available to researchers, caregivers, or policymakers in that trial results are often not reported by sex [221] (Fig. 4). Especially in view of requirements for inclusion of women in clinical trials, several recent studies have highlighted the importance of such reporting by emphasizing the clinical differences between sexes in the response to medications [222–224]. In addition, the side effect profile of pharmacologic agents may differ by sex, specifically, women are more likely to suffer torsades de pointes and increased bleeding [225, 226] compared with men. Thus, strong scientific and clinical concerns suggest encouraging, if not requiring, that trials and research sponsors be obligated to separately report data generated in women. Moreover, this could also be accomplished by a requirement by journal editors to include sex-specific reporting in all primary manuscripts.

### FDA requirements

In the Food and Drug Administration Safety and Innovation Act (Public Law No. 112-144) signed into law 9 July 2012 required demographic data and analysis of sex, age, race, and ethnicity to be posted in a report on the FDA website within a year of enactment and an action plan to be developed. In December 2011, the FDA issued a “Draft Guidance for Industry and Food and Drug Administration staff,” on “Evaluation of Sex Differences in Medical Device Clinical Studies.” This FDA document addresses the scope of the problem, the importance of considering sex differences, barriers to enrollment of women, and recommendations for achieving representative enrollment. This guide recommends for the disease or condition for which the device is intended to treat or diagnose: sex-specific prevalence, sex-specific diagnosis and treatment patterns; identification of proportions of women included in past studies for the target indications; and identification of any known clinically significant sex differences in outcomes related to either safety or effectiveness. The recommendation was that this information be included in the study and submission documents for (1) new or ongoing studies, (2) completed studies (marketing application stage), and (3) post-market studies (PHS 522 PS stage). In addition, altering pre-market investigational paradigms regarding sex-based data were encouraged by alignment of regulatory requirements and enhancement of business incentives for this purpose [207]. Included among the paradigms considered was that a discussion about the impact of sex should be undertaken before devices or drugs receive FDA approval. Tactics discussed included (1) inclusion of sex on the checklist for document preparation for FDA-sponsored meetings, (2) mandatory substantive review of available sex-specific data with each phase 2 report with obligatory inclusion in meeting minutes, and (3) amending ICH E3 guidelines. Similar requirements to tighten FDA regulations for reporting sex- and gender-based data about new and experimental medicines and devices were included in the Heart Disease Education, Analysis and Research, and Treatment (HEART) for Women Act proposed legislation introduced to the 112th Congress (2011–2012).

Another suggestion with broad implications was the consideration of a new policy allowing extensions in patent duration for additional research in women such as those granted for new pediatric indications, which have been highly successful in encouraging research on medications in children. Advocacy groups were encouraged to add technical expertise to better address these issues within their current missions. Finally, strategies to increase awareness of the problem among investigators, industry, and regulators have been endorsed, including requiring investigators to complete continuing medical education on sex differences in disease, multidisciplinary forums such as those held by the Drug Information Agency, educational seminars with FDA leadership, and increased use of existing NIH online educational materials [16].

### Adverse event (AE) and serious adverse event (AE) reporting

Improving the accessibility of new and existing data for review and for incorporation into meta-analyses is needed [227]. AE and SAE data stratified by sex should be included in the primary or secondary manuscript and/or available in an appendix to the primary manuscript. Secondary presentations and/or papers on results in women or sex comparisons should be encouraged by conference chairs, editors, and academic institutions. Alternative ways should also be explored to make these data publicly available, such as online appendices. The American Heart Journal has already taken a public stand in favor of such reporting [16, 228]. Other novel approaches to improve data availability in women include enhancing the bioinformatics infrastructure to standardize electronic clinical data (Clinical Data Interchange Standards Consortium http://www.cdisc.org) to allow tracking of recruitment, analyzing data across clinical trials that alone do not have sufficient numbers of women for meaningful analyses, and analyzing of adverse events. Recently, the FDA has embarked on an ambitious bioinformatics modernization effort that includes an effort to harmonize with the standards being used in exchange of Electronic Health Records, which may improve our ability to detect important sex differences [227].

### Statistical analysis: how to analyze by sex, what needs to be considered to analyze in sex-specific fashion, analyze adjusting for sex, and analyzing for interaction by sex

A variable for male or female is usually collected in clinical study datasets. We advise that a clear definition of the term is provided for each study. Sex is the biologic variable and gender a self-declared variable encompassing psychosocial and cultural attributes. A primary consideration for sex-specific analyses is that if sex or gender is an a priori secondary analysis, then the enrolled cohort of women should be sufficiently powered for comparative analysis of the primary aim. Without this a priori planning, all reported sex and gender comparisons should include a post hoc statistical power calculation. In treatment trials, differential procedural effectiveness and complications as well as adherence to guideline-directed lifestyle and medical therapy should be reported as clinical management recommendations are often suboptimal among women as compared to men. Appendices reporting a more detailed analysis by sex/gender, including subsets (e.g., younger women) should be included for all major observational and clinical trials. Consideration should also be given to targeted population strata of women of diverse race and ethnicity as these subsets report the highest morbid and fatal event rates. In many cases, adjusted analyses are presented that control for clinical and comorbid covariates that may minimize sex differences. It is recommended that absolute rates of morbid and fatal event rates also be included with adjusted analyses. Care should be taken when women are matched to subsets of men by a priori chosen criteria (e.g., propensity matching techniques). Matching women to men may select a subset of atypical females and minimize unique sex-specific differences. If matching is performed, details as to how the selected women compared to the whole subset of females should be provided as well as additional sex stratified analyses.

## Conclusions

Efforts to develop and use sex-specific information regarding women have increased over the last two decades, and while there is still a great need for more data, the evidence base has increased. We encourage investigators to consider these variables that have impact on women’s CVD risk and outcomes when designing studies. Table 4 provides a summary of variables to consider and a sampling of proposed questions to document pregnancy, menopause, and psychosocial variables. Programs that involve women across their lifespan should also consider the participants’ future risk for CVD risk. Incorporation of sex-specific knowledge into research strategies is aimed at improving clinical CVD outcomes and addressing health disparities for women.

**Table 4:** Variables to consider in studies of woman’s cardiovascular disease and risk

**General risk factors**	**Information to capture**
Age	
Height/weight	BMI
Waist circumference	
Smoking status	Prior, current
History of hypertension	Blood pressure
Blood lipids (HDL, LDL, triglycerides)	
Diabetes, glucose, insulin,	
Inflammation: hsCRP	
History of chronic inflammatory disease	(Asthma, inflammatory rheumatologic disease, migraine, inflammatory bowel disease)
Prior cancer	Type (breast, etc), chest radiation, chemotherapy
Prior CVD	Angina, myocardial infarction, cerebrovascular disease, coronary revascularization procedures, peripheral arterial disease, heart failure
**Sex-specific or less conventional risk factors**	
**Pregnancy-related variables**	**Questions to ascertain information**
Parity	Number of pregnancies lasting >20 weeks
Fetal deaths	Number of miscarriages <20 weeks, stillbirths
History of preeclampsia	Have you ever had preeclampsia or toxemia?
History of gestational hypertension	Have you ever had gestational hypertension (pregnancy-related high blood pressure or pregnancy-induced hypertension)?
History of gestational diabetes	Have you ever had gestational diabetes (new onset diabetes of pregnancy)?
Offspring birthweight and gestation length (when assessed together, this allows calculation of small-for-gestational age and large-for-gestational age)	Birthweight of each child (lbs and ounces) and gestation length:
Low birthweight	Have you ever delivered an infant weighing less than 5 lbs 8 oz (less than 2500 g)?
Macrosomia (indicative of gestational diabetes)	Have you ever delivered an infant weighing more than 10 lbs (more than 4500 g)?
**Menopause-related variables**	**Questions to ascertain information**
Menopausal status	Have your natural menstrual periods ceased permanently? (No; Yes—no menstrual periods; Yes—had menopause but now periods induced by hormones; Not sure)
	At what age did natural periods stop?
	For what reason? (natural; surgical; radiation or chemotherapy; others)
	Did you have a hysterectomy, if so at what age
	Did you have removal of ovary (unilateral or bilateral) and if so, at what age
Current use of hormones	Are you currently using:
	- Oral contraceptives,
	- Transdermal hormone therapy
	- Vaginal hormone therapy
	Have you ever used these therapies?
Menstrual regularity	What is the current usual pattern of your menstrual cycles (when not pregnant, lactating, or on the pill): extremely regular (no more than 1–2 days before or after expected); very regular (within 3–4 days); regular (within 5–6 days); usually irregular; always irregular; no periods
**Psychosocial Variables**	**Questions to ascertain information**
History of violent abuse	Before age 18, did any adult in your family:
	- Push, grab, or shove you
	- Kick, bite, or punch you
	- Hit you with something that hurt your body
	- Choke or burn you
	- Force you into sexual activity by threatening you, holding you down, or hurting you in some way when you did not want to
	- Physically attack you in some other way
	Responses: never; once; a few times; more than a few times
	Since age 18, has anyone (repeat above)
Current depression screener	Clinical screener recommended by USPSTF:
	- Over the past 2 weeks, have you felt down, depressed, or hopeless?
	- Over the past 2 weeks, have you felt little interest or pleasure in doing things?
	Antidepressant use (e.g., Prozac, Zoloft, Lexapro, Pamelor, Cymbalta)
	More formal screening tools include:
	- Beck Depression Inventory
	- General Health Questionnaire
	- Center for Epidemiologic Study Depression Scales (CES-D)
	- Patient Health Questionnaire PHQ 9 (Quick Depression Assessment)
History of depression screener	In your lifetime, have you ever had 2 weeks or longer when nearly every day you felt sad, blue, or depressed for most of the day?
	Did you ever tell a doctor or mental health specialist that you were feeling depressed?
	Has a health provider ever diagnosed you with depression?
Current psychosocial stress	Short version of Cohen Perceived Stress Scale:
	In the last month, how often have you
	- felt that you were unable to control the important things in your life?
	- felt confident about your ability to handle your personal problems?
	- felt that things were going your way?
	- felt difficulties were piling up so high that you could not overcome them?
	Reponses: never; almost never; sometimes; fairly often; very often
